# CNNSplice: Robust models for splice site prediction using convolutional neural networks

**DOI:** 10.1016/j.csbj.2023.05.031

**Published:** 2023-05-30

**Authors:** Victor Akpokiro, H. M. A. Mohit Chowdhury, Samuel Olowofila, Raisa Nusrat, Oluwatosin Oluwadare

**Affiliations:** Department of Computer Science, University of Colorado, Colorado Springs, CO 80918, United States

**Keywords:** Splice sites (SS), Deep Learning (DL), Convolutional neural network (CNN), Dense Neural network (DNN), Feature extraction

## Abstract

The identification of splice site, or segments of an RNA gene where noncoding and coding sequences are connected in the 5′ and 3′ directions, is an essential post-transcriptional step for the annotation of functional genes and is required for the study and analysis of biological function in eukaryotic organisms through protein production and gene expression. Splice site detection tools have been proposed for this purpose; however, the models of these tools have a specific use case and are inefficiently or typically untransferable between organisms. Here, we present CNNSplice, a set of deep convolutional neural network models for splice site prediction. Using the five-fold cross-validation model selection technique, we explore several models based on typical machine learning applications and propose five high-performing models to efficiently predict the true and false SS in balanced and imbalanced datasets. Our evaluation results indicate that CNNSplice’s models achieve a better performance compared with existing methods across five organisms’ datasets. In addition, our generality test shows CNNSplice’s model ability to predict and annotate splice sites in new or poorly trained genome datasets indicating a broad application spectrum. CNNSplice demonstrates improved model prediction, interpretability, and generalizability on genomic datasets compared to existing splice site prediction tools. We have developed a web server for the CNNSplice algorithm which can be publicly accessed here: http://www.cnnsplice.online

## 1 Introduction

**Fig. 1:**
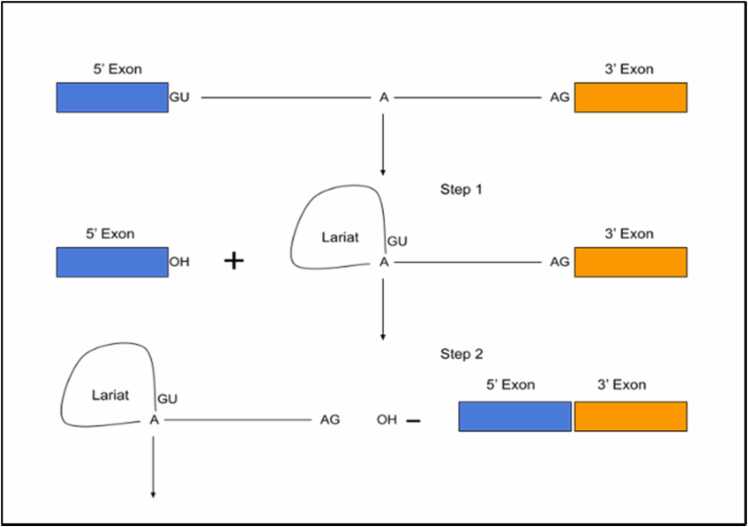
Analysis of the splice sites biochemical process. This figure shows the two-stage biochemical process for splice site and sequence distribution in splice site location. The OH represent the 3′ hydroxyl group of an RNA base.

Gene annotation is essential to understanding the biological function of the genomic sequence in living organisms [1]. RNA splicing—a subtask of gene annotation—involves removing the noncoding regions (introns) and joins the protein-coding regions (exons) in the eukaryotic gene, which in turn is required for gene expression and protein synthesis comprehension. The transition points between the exons and introns are called splice sites (SS). This splicing process, which occurs during gene transcription, involves converting pre-mRNA to mRNA to extract crucial information from the nucleus and express it (translated into actual protein). RNA splicing is the term for this process. Eukaryotes have huge genome sizes and small exons that are surrounded by large introns [1]. In the direction 3′ to 5′ of the intron downstream, the consensus AG dinucleotide sequence is expressed as an acceptor SS, and in the direction 5′ to 3′ of the intron upstream, the consensus GT sequence is expressed as a donor SS [2], [3], [4]. Acceptor and donor SS with AG and GT dinucleotide pairings make up the salient amount of SS in a genome and are both known as canonical SS [5], with noncanonical SS also observed. The canonical sequence distribution in an SS location, as well as intron splicing, are depicted in Fig. 1 as a pictorial representation of the SS biochemical process. Thus, many fast and efficient computational-based approaches/algorithms have been developed for SS prediction, and these algorithms can be categorized into subgroups such as the machine learning approach [6], [7], [8], which utilizes nonlinear transformation to perform feature extraction by learning patterns from consensus AG/GT dinucleotide combination and their surrounding molecules; information gain theory [9], [10], [11], which measures the degree of mixing of classes for all samples and any position in the nucleotide sequences; probabilistic measure [12], [13], which computes the maximum likelihood from the probability of a specific SS position; and discriminant analysis approach [14], which applies the statistical measure to determine a particular position of consensus AG/GT nucleotide sequence. The extraction of features or consensus patterns from a group of nucleotide sequences to detect or create links between SS and their surrounding regions is a common element of these approaches. Of all these method subgroups, two machine learning-based algorithms—deep learning (DL) and support vector machine (SVM)—offer more optimal approaches, including shorter prediction time with increased accuracy, and have recently been a prominent approach to SS prediction. To predict SS, many SVM models use a combination of Markov models (MM) and maximum dependency decomposition (MDD) [13], [15]. GeneSplicer [16] integrated the MM with MDD, which was reported by Burge et al. [13] to improve their SS prediction. Pashaei et al. [17] investigated the effectiveness of third-order Markovian encoding models and SVMs in predicting human SS. They also proposed a novel method for SS prediction that utilizes sequence component analysis and hidden Markov model (HMM) [18], which they demonstrated to outperform existing methods in accurately predicting SS in genomic DNA.

While the sophistication of these machine learning models increased, their accuracy slowly improved. This resulted from the computational time concerns and the ineffective manual feature selection and extraction from the input variables. Hence, convolutional neural network (CNN) architectures have been used more frequently in SS prediction work using the DL approach, with varying depth, parameters, and architecture design [19], [20], [21], [22], [23]. Albaradei et al. [21] developed Splice2Deep, a deep CNN ensemble model that predicts SS in cross-organisms and offers the ability for new SS genome organism annotation. Similarly, Zuallaert et al. [20] developed SpliceRover, a model that uses CNN to predict SS. They demonstrated the interpretability of their model by showing genomic information relevant to SS detection. Wang et al. [24] developed SpliceFinder, an SS tool that predicts canonical (acceptor and donor) and noncanonical SS using a one-layer CNN. Also, Akpokiro et al. [25] developed DeepSplicer, a CNN model using five-fold cross-validation and three convolutional layers of CNN architecture to predict SS in genomic organism datasets.

Although the DL-based algorithms for SS prediction have seen significant advancements, there is still a need for more performance improvements especially because many of these tools have an imbalanced performance across organisms for SS detection. Specifically, they are inflexible and not comprehensive in maintaining a consistent prediction accuracy when used for SS detection across a wide range of organisms. Also, the models of some of the existing methods are not robust to the data distribution of acceptor and donor SS made available to them for training and testing purposes, as we show in detail in our results section; thus, their detection accuracy performance varies depending on the acceptor and donor sites’ datasets ratio used for the algorithm’s training. Hence, we propose CNNSplice, a set of DL-based models, for detecting SS in organisms using robust CNN algorithm architectures. For SS prediction, CNNSplice models are trained using a preselected set of organismic datasets. The models in this study were chosen based on the cross-validation results on the datasets. According to our evaluation results, CNNSplice enhances SS detection research by producing highly effective SS detection models (Models 1, 2, 3, 4, and 5), as well as a model (Model 1) that can accurately predict or annotate newly sequenced datasets.

## 2 Material and methods

### 2.1 Datasets

We created a balanced and imbalanced dataset from five carefully selected datasets of organisms, namely: *Homo sapiens*, *Oryza sativa japonica*, *Arabidopsis thaliana*, *Drosophila melanogaster*, and *Caenorhabditis elegans*. The reference genomic sequence data for these organisms were obtained from Albaradei et al. (2020) [21], and separate acceptor and donor models were generated from each organism dataset. The outcome of this method yielded a balanced dataset consisting of confirmed true and false regions at a 1:1 ratio, totaling 10,000 entries each. Additionally, an imbalanced dataset with a confirmed true-to-false region ratio of 3:1 was generated, totaling 7500 true and 2500 false regions, with 10,000 entries in total, as detailed in Table 1. For the imbalanced dataset, we also utilized an empirical sliding window program to effectively select the local flanking regions and the SS-containing region based on the sequence distribution ratio; the same applied for the balanced dataset. The relevance and justification for this dataset split is twofold, especially concerning the introduction of the imbalanced dataset. First, it was to assess the impact of the training dataset distribution on our algorithm’s performance. Second, it was to determine and develop a robust model for SS prediction across multiple organisms. This allows us to assess the strength of our models in scenarios where they are used to detect SS from datasets without knowing the data distribution, which is the case for real-life use scenarios of the SS detection tools. Hence, we propose a model that is not overly influenced by the training dataset and would perform well in extreme cases where the distribution of false or true cases is skewed.

**Table 1:** The dataset’s sequence count.

Splice sites	Distribution	Sequence region	Distribution ratio
Acceptor (AcSS)	Balanced	5000 (true) 5000 (false)	1:1
	Imbalanced	7500 (true) 2500 (false)	3:1
Donor (DoSS)	Balanced	5000 (true) 5000 (false)	1:1
	Imbalanced	7500 (true) 2500 (false)	3:1

This shows the dataset distribution, sequence counts, and distribution ratio for balanced and imbalanced acceptor and donor sequenced organisms. The balanced datasets are evenly distributed, and the imbalanced datasets are distributed in the ratio of 3–1 (true to false).

### 2.2 One-hot encoding and parameter tuning

**Fig. 2:**
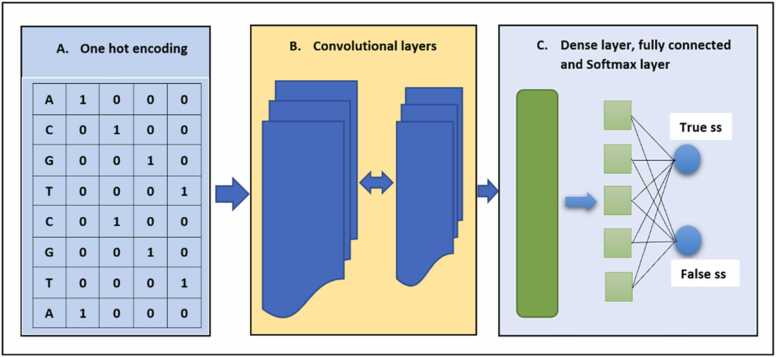
CNNSplice architectural pipeline. This figure shows the CNNSplice pipeline with each block representing Fig. 2 A, Fig. 2B, and Fig. 2 C respectively. Fig. 2 A shows the one-hot encoding as described in the one-hot encoding and hyper-parameter tuning section, Fig. 2B depicts the convolutional layer and Fig. 2 C shows the output layers which includes the dense, the fully connected layer, ss denotes splice site.

One-hot encoding, which involves converting categorical data to numerical data variables for improved machine learning algorithm prediction, is one of the aspects of data preprocessing in DL. One-hot encoding is typically applied to categorical data, whereby each category is expressed as a binary vector containing a single 1 with all other elements as 0. This encoding technique is particularly advantageous when there is no inherent order to the categories, and they are mutually exclusive. Additionally, CNNs can effectively learn to identify distinctive patterns and characteristics that correspond to each category through the use of one-hot encoded input. In this study, we utilized a binary integer variable collection to represent our input genomic nucleotide bases. Specifically, we assigned [1 0 0 0], [0 1 0 0], [0 0 1 0], and [0 0 0 1] to represent Adenine (A), Cytosine (C), Guanine (G), and Thymine (T), respectively. Additionally, we represented invalid nucleotide letters (N) with [0,0,0,0], where each nucleotide is located at the corresponding vector index containing a value of 1. Therefore, we provided the CNN architecture with a Z X 4 input matrix, where Z denotes the genomic sequence length and 4 denotes the nucleotide types (A, C, G, T). Fig. 2*A* shows a pictorial illustration of the one-hot encoding. We tuned the hyperparameters on our generated hyperparameter datasets—to avoid bias in CNN splice—during learning based on the search space shown in Table 2 and the selected ranges for the model generation. We fine-tuned the hyperparameters and chose the top-performing parameters based on the minimum validation loss.

**Table 2:** The grid search space table for the tuning of the Convolutional neural network hyperparameters.

Hyper-Parameters	Search Space	Selected Ranges
Layers	[1, 2, 3, 4, 5]	[1, 2, 3]
Filter number	[50,64,100,128,150]	[5]
Filter size	[3,5,7,9,11]	[3, 9]
Stride	[1,3,5]	[1]
Activation function	[sigmoid, relu, softmax, tanh]	[sigmoid, relu]
Learning rate	[1e-3, 5e-4, 1e-4, 5e-5, 1e-5]	[1e–4]
Optimizers	[sgd, adam, adagrad, RMSprop, nadam]	[adam]

This table shows different hyper-parameter lists from which the CNN architecture was tuned to select the best parameters for the model’s configuration.

### 2.3 Convolutional neural network model

The CNN contains the convolutional layers, which are the main building block of this neural network [26]. These convolutional layers are made up of neurons that contain and perform element-wise multiplication operations between the inputs and the weights. To employ a reliable model, each CNNSplice model has different convolutional layers (Fig. 2*B*). These convolutional layers perform local and global feature extraction on the acceptor and donor genomic input sequence. Importantly, the convolutional layer consists of a complex set of learnable filters with varying filter length and depth, which enables efficient discrimination of true/false acceptor and donor SS. The best-performing model has been selected based on the average validation accuracy across the preselected organism for acceptor and donor individually. In the cross-validation section, we presented 11 architectures from which the consistent top-performing models were selected as the architecture for the convolutional layer of our pipeline.

Each model convolutional block contains a flattened layer, a fully connected layer of 100 neurons. By using all its connections to all the activations in the flatten layer, the fully connected layer increases nonlinearity expression capabilities to detect both canonical and noncanonical SS and improve CNNSplice’s generalization ability (Fig. 2*C*). Their activations may be approximated using matrix multiplication and bias offset. This layer uses the ReLU [27] activation function, which uses element-wise nonlinearity. To avoid overfitting, a 30 % dropout [28] layer is implemented. Finally, we used the Adam optimizer with a learning rate of 1e-4, cross-entropy for the loss function, and the Softmax activation function layer [29] to transform the prediction output to normalized probability. A visual summary of the models is shown in Fig. 2.

### 2.4 Genomic datasets sequence length selection

Statistical examination of the correlation between genomic sequence length and prediction performance has been done in the previous SS prediction research [19], [21], [24] because the sequence length of the produced dataset is an important factor to consider for SS prediction. Experiments by Wang et al. [24] and Du et al. [19] show that the capacity of SS models to predict rises as the length of the genomic sequence increases. Though there is a favorable association between sequence length and prediction performance, a long sequence length may degrade classification performance [16], [20], [21]. To maintain a good sequence length balance, and for a fair model prediction and comparison with other baseline methods, we used a sequence length of 400 of sequence region 0–399. Thus, all algorithm acceptor and donor input species datasets regions in this study have a sequence length of 400 nucleotides bases. The dataset section clearly details the source, distribution, and the true and false AcSS/DoSS regions for each dataset used in the experiment.

### 2.5 Evaluation metrics

We used the following metrics to evaluate the performance of our neural network models.

• The Recall evaluates as:
  (1) $$\mathrm{Recall}=\frac{\mathrm{TP}}{\;\mathrm{TP}+\mathrm{FN}\;}$$
  where TP is the number of true positives, FN is the number of false negatives.
• The Precision evaluates as:
  (2) $$\mathrm{Precision}=\frac{\mathrm{TP}}{\mathrm{TP}+\mathrm{FP}\;}$$
  where TP is the number of true positives, FP is the number of false positives.
• The Accuracy evaluates as:

(3) $$\mathrm{Accuracy}=\frac{\mathrm{TP}+\mathrm{TN}}{\mathrm{TP}+\mathrm{TN}+\mathrm{FP}+\mathrm{FN}}$$

## 3 Results

### 3.1 Cross-validation

To get a smoother, less noisy, and more sustainable estimate of how well a model performs, cross-validation, which involves iterating over a K-fold validation set and determining the average across the datasets, is essential. K-1 fold is reserved for training purposes with one-fold for testing. In summary, cross-validation is used to return partitions of the dataset for training and evaluation of the model for enhanced neural network performance and statistical probability. CNNSplice splits the training dataset into five folds using K-fold cross-validation with the StratifiedKFold machine learning module [19]. We reconstructed the cross-validation datasets from the source datasets [21]. The training dataset, X, is split into five equally distributed subset datasets with the cross-validation computation producing a mean output of the five model accuracies and losses from five data splits. This can be represented mathematically as follows:

(4) $$X=X_{1}\cup X_{2}\cup X_{3}\cup X_{4}\cup X_{5}$$

Where X is the entire training dataset and is split into equal subsets for all *i* = 1, 2, 3, 4, 5. Eq. 4 yields five subsets from dataset X. Mathematically, the process of cross-validation is given by

(5) $$|X_{1}|\approx {|X}_{2}|\approx {|X}_{3}|\approx |X_{4}|\approx |X_{5}|$$

As shown in Table 1, we constructed 5000 true and 5000 false acceptor and donor datasets for the balanced experiments and 7500 true and 2500 false acceptor and donor datasets for the imbalanced experiment evenly. To find the best-performing convolutional layer for Fig. 2B, we performed cross-validation on the list of potential architectures as follows:

• **C1**: is a one-dimensional convolutional layer block.
• **C11**M: has a pair of one-dimensional convolutional layer and max-pooling layer.
• **Model** 1: contains three (3) blocks of one-dimensional convolutional layer, and a max-pooling layer for each convolutional block.
• **Model** 2: contains two (2) layers of a pair of one-dimensional convolutional layer and max-pooling layer.
• **NPOOL1**3: is similar to Model 1 and Model 3 except it has no max-pooling or average-pooling layer to each convolutional block.
• **Model** 3: contains three (3) blocks of a pair of one-dimensional convolutional layer and average pooling layer.
• **MEANPOOL1**3: is similar to NPOOL13 with a one-dimensional global average pooling layer connected in the fully connected layer.
• **Model** 4: averages the weight generated from Model 1 and Model 2.
• **Model** 5: involves weights from Model 1 and Model 3 averaged.
• **NPOOL1**2: is similar to Model 2 except it has no max-pooling or average-pooling layer to each convolutional block.
• **AVEPOOL1**2: contains two (2) layers of a pair of one-dimensional convolutional layer and average-pooling layer.

As shown in Fig. 2, each of the aforementioned architectures is the variations of the architecture used in Fig. 2*B*, which accepts one-hot encoding as input. The outputs of each of these architectures, in Fig. 2*B*, serve as input to the dense and fully connected layer as seen in Fig. 2*C*. Cross-validation was performed on all the aforementioned architectures for the five organisms’ balanced and imbalanced datasets.

Our work is focused on the development of robust models that are capable of high performance on multiple organisms. Thus, to select the best-performing models, we averaged the results generated by each model across the different organisms to identify which models have a consistently high performance. We have shown the tabular results of the cross-validation experiment in Table 3 and Table 4 for the balanced and imbalanced datasets results respectively. Across the five organisms’ acceptor and donor datasets for the imbalanced and balanced datasets we created, Model 1, Model 5, and Model 3 are the top three (3) performing model architectures for balanced acceptor datasets; Model 1, Model 5, and Model 4 are the top three (3) performing model architectures for balanced donor datasets; Model 1, Model 2, and Model 5 are the top three (3) performing model architectures for imbalanced acceptor datasets; and Model 1, Model 2, and Model 4 are the top three (3) performing model architectures for imbalanced donor datasets.

**Table 3:** The cross-validation results for the Balanced Dataset for the five organisms.

This Table depicts the 5-fold Cross-validation Results, average result across the organism distribution, and average results positions for Balanced datasets across the selected organism in percent (%). With respect to validation accuracy, results highlighted shows that * represent the best result * * represents the second best, * ** represents the third. HS denotes Homo sapiens, Oryza denotes *Oryza sativa japonica*, AT denotes *Arabidopsis thaliana*, D. Mel denotes *Drosophila melanogaster*, and C. elegans denotes *Caenorhabditis elegans*.

**Table 4:** The cross-validation results for the imbalanced dataset for the five organisms.

This Table depicts the 5-fold Cross-validation Results, average result across the organism distribution, and average results positions for Imbalanced datasets across the selected organism in percent (%). With respect to validation accuracy, results highlighted shows that * represent the best result * * represents the second best, * ** represents the third. HS denotes Homo sapiens, Oryza denotes *Oryza sativa japonica*, AT denotes *Arabidopsis thaliana*, D. Mel denotes *Drosophila melanogaster*, and C. elegans denotes *Caenorhabditis elegans*.

**Fig. 3:**
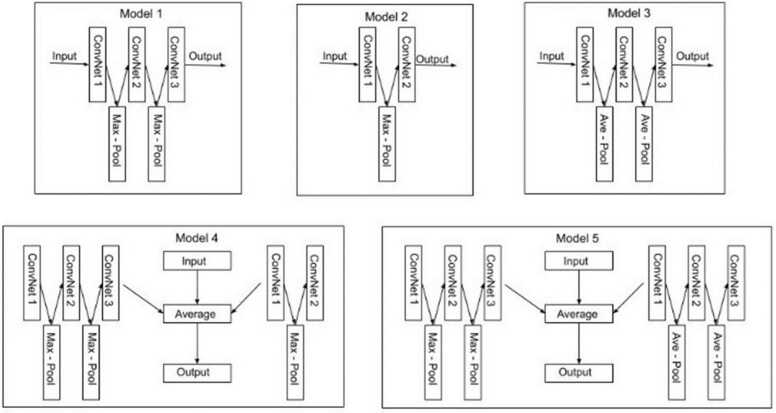
Best performing CNNSplice model architectures. This figure shows a detailed layout of the CNNSplice models’ convolutional block architecture selected by the 5-fold cross validation process for Fig. 2B of the CNNSplice pipeline illustration, Fig. 2. ConvNet means the convolutional neural network layer, Max - Pool is the max-pooling layer, and the Ave - Pool is the average pooling layer. The input block denotes Fig. 2 A and the output block denotes Fig. 2 C.

Hence, based on the results obtained from the cross-validation experiment as shown in Table 3 and Table 4, the architectural formation of the five best-performing models based on average accuracy across the organisms is described as follows, with a schematic representation shown in Fig. 3.

• **Model 1:** This model has three (3) one-dimensional convolutional layers, each with 50 kernels of size 9 and a batch size input of *L X 4*, the sequence length, where L is the sequence length. ReLU activation function is applied to each convolutional layer. In addition, a max-pooling layer of pool size 2 and stride 1 is applied to each individual convolutional layer.
• **Model 2:** This model has two (2) one-dimensional convolutional layers, each with 50 size 9 kernels and a batch size input of L X 4, where L is the sequence length. ReLU activation function is applied to each convolutional layer. In addition, a max-pooling layer of pool size 2 and stride 1 is applied to each individual convolutional layer.
• **Model 3:** This model has three (3) one-dimensional convolutional layers, each with 50 kernels of size 9 and a batch size input of *L X 4*, the sequence length, where L is the sequence length. ReLU activation function is applied to each convolutional layer. In addition, an average pooling layer of pool size 2 and stride 1 is applied to each individual convolutional layer.
• **Model 4:** This involves setting a new weight obtained from the mean weight across a set weight from Model 1 and Model 2 to the model.
• **Model 5:** This involves setting a new weight obtained from the mean weight across a set weight from Model 1 and Model 3 to the model.

### 3.2 Benchmark methods for model comparison

In the context of SS prediction using the DL approach, various CNN architectures of different depths, parameters, and architecture designs have been used. Herein, we describe our benchmark cutting-edge models for comparison.

**Wang, R. et al. (2019)**.

Wang et al. (2019) proposed SpliceFinder [24], a CNN-based model that effectively predicts SS in various species, achieving a classification accuracy of 90.25 % in the human dataset. We chose this recent study as our baseline model because it can be applied to annotate new species without the need for retraining.

**Albaradei, S. et al. (2020)**.

Albaradei et al. (2020) introduced Splice2Deep [21], a high-performing SS prediction tool that uses deep CNNs and achieves high accuracy in various datasets, including *Homo sapiens*, *Oryza sativa japonica*, *Arabidopsis thaliana*, *Drosophila melanogaster*, and *Caenorhabditis elegans*. The authors also demonstrated the interpretability of the model by identifying important regions and motifs for SS prediction, as well as performing cross-organism validation test.

**Zuallaert, J. et al. (2018)**.

Zuallaert et al. (2018) proposed SpliceRover [20], a DL method that predicts SS using interpretable CNNs. The authors demonstrated the effectiveness of SpliceRover on multiple datasets and species, achieving up to 80.9 % accuracy. For benchmarking, we used their publicly available web server found at http://bioit2.irc.ugent.be/rover/splicerover.

**Pertea, M. et al. (2001)**.

Pertea et al. (2001) presented GeneSplicer [16], a computational approach to SS prediction that combines MM technique and MDD to identify SS in DNA sequences. The authors showed that GeneSplicer achieves high accuracy in predicting SS in various organisms, including human, mouse, and rat genomes. For benchmarking, we used their publicly available web server found at https://www.cbcb.umd.edu/software/GeneSplicer/gene_spl.shtml.

**Akpokiro, V. et al. (2021)**.

Finally, Akpokiro et al. (2021) proposed DeepSplicer [25], a DL-based method for predicting SS in DNA sequences. Similar to CNNSplice, DeepSplicer performed a five-fold cross-validation test and achieved high accuracy in various datasets, including *Homo sapiens*, *Oryza sativa japonica*, *Arabidopsis thaliana*, *Drosophila melanogaster*, and *Caenorhabditis elegans*. The data, models generated, and source code for DeepSplicer [25] and SpliceFinder [24] are all available in their respective GitHub repositories.

### 3.3 Model evaluation and performance comparison

**Fig. 4:**
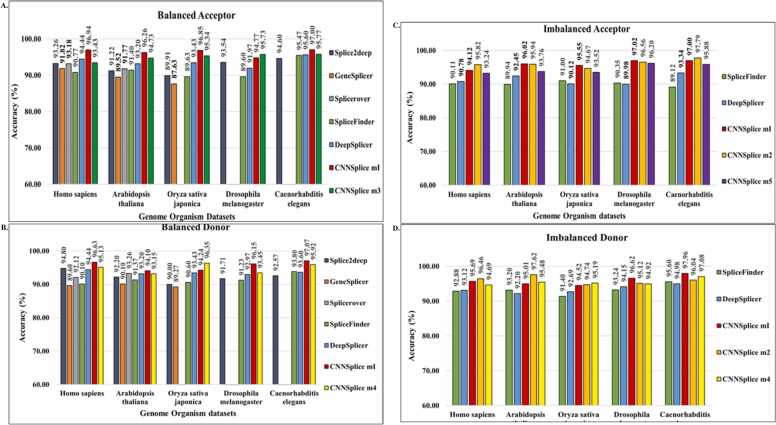
A comparison of the performance of CNNSplice models and other models for (A) balanced acceptor, (B) balanced donor, (C) imbalanced acceptor, and (D) imbalanced donor datasets. This chart shows the CNNSplice models comparison to the models based on the performance accuracy in predicting splice sites for balanced acceptor datasets. CNNSplice m1 indicate Model 1, CNNSplice m2 indicate Model 2 CNNSplice m3 indicates Model 3, CNNSplice m4 indicate Model 4, CNNSplice m5 indicate Model 5. For this plot’s label, the Y-axis represents the accuracy in percentage (%) as the X-axis represents the name of the organisms’ datasets.

We built separate models from our CNN algorithm architecture to reliably predict acceptor and donor SS for each of the balanced and imbalanced datasets. To improve the prediction performance of our selected models we performed feature extraction along the three regions: two local opposite genome surrounding regions (upstream and downstream) and the SS surrounding region [21]. These models were selected based on peak performance from evaluating the mean prediction accuracy results across the preselected organism sequence datasets on the five-fold cross-validation results. Following feature extraction on each dataset, dinucleotides discovered on SS contain AG for acceptor sites and GT for donor sites; this AG-GT result consensus confirms previous observations in scientific literature [19], [21], [25], [30]. It is crucial to highlight that we chose our models based on the outcomes of our cross-validation experiments, and we fairly compared them—as shown in Fig. 4*a*, Fig. 4*b*, Fig. 4*c*, and Fig. 4*d*—to the aforementioned state-of-the-art methods by using our test dataset with the models provided by the authors, as well as for training and testing in cases where the model was unavailable but the method architecture was available with our datasets.

These figures indicate that Model 1 consistently provided a high prediction accuracy across all acceptor and donor organisms for both datasets—balanced and imbalanced. In addition to Model 1, Model 3 performed well in balanced acceptor datasets (Fig. 4*a*), Model 2 and Model 5 performed better in imbalanced acceptor datasets (Fig. 4*c*), Model 1 and Model 4 performed better in balanced donor datasets (Fig. 4*b*), and Model 2 and Model 4 performed better in imbalanced donor datasets (Fig. 4*d*). In addition, we compared our models’ performance to the state-of-the-art SS prediction algorithms—Splice2Deep [21], GeneSplicer [16], SpliceRover [20], SpliceFinder [24], DeepSplicer [25]—on the five organisms’ datasets: *Homo sapiens*, *Oryza sativa japonica*, *Arabidopsis thaliana*, *Drosophila melanogaster*, and *Caenorhabditis elegans* organisms (Fig. 4*a*–Fig. 4*d*). Model 1 achieved the highest performance accuracy compared to the other models and the state of the art for the balanced acceptor *Homo sapiens*, *Arabidopsis thaliana*, *Oryza sativa japonica*, and *Caenorhabditis elegans* organisms’ dataset with 96.94 %, 96.26 %, 96.85 %, and 97.00 % accuracy respectively (Fig. 4*a*). For the balanced donor dataset, Model 1 performed better than the other models and the Splice2Deep [21], GeneSplicer [16], and SpliceFinder [24] models for *Homo sapiens*, *Arabidopsis thaliana*, *Drosophila melanogaster*, and *Caenorhabditis elegans* organisms with accuracy performance of 96.63 %, 94.10 %, 96.15 %, and 97.07 % respectively (Fig. 4*b*). Model 4 performed highest for the *Oryza sativa japonica* organism dataset with an accuracy of 96.35 % (Fig. 4*b*).

Also, we investigated the SS prediction potentials with imbalanced acceptor and donor organism datasets and compared our model’s performance with state-of-the-art models—SpliceFinder [24] and DeepSplicer [25]. It is worth noting that for this analysis, we benchmarked only with the SpliceFinder [24] and DeepSplicer [25] algorithms because the other state-of-the-art methods examined previously do not have available models trained with imbalanced organisms’ datasets or do not provide model architecture for model retraining with imbalanced datasets. Model 2 performed better than the DeepSplicer [25] and SpliceFinder [24] for imbalanced acceptor organisms’ datasets of *Homo sapiens* and *Caenorhabditis elegans* with performance accuracy of 95.82 % and 97.79 % respectively, whereas Model 1 performed better for the *Arabidopsis thaliana*, *Oryza sativa japonica*, and *Drosophila melanogaster* organism datasets with an accuracy of 96.02 %, 95.55 %, and 97.02 % respectively (Fig. 4*c*). For the imbalanced donor, Model 1 performed better for *Drosophila melanogaster* and *Caenorhabditis elegans* organisms with SS prediction accuracy of 96.62 % and 97.96 % respectively, Model 2 had a prediction accuracy of 96.46 % and 97.62 % for *Homo sapiens* and *Arabidopsis thaliana* respectively, and Model 4 had a prediction accuracy of 95.19 % for the *Oryza sativa japonica* organism dataset (Fig. 4*d*). We present tables showing the results and comparison with other methods in Table 5 and Table 6. To show the performance evaluation metrics for CNN Splice, we show in Table 7 the results of the precision, recall, F1, and accuracy evaluation metrics for the Model 1 test prediction using the balanced genomic datasets. We used N/A to indicate methodologies that did not use the considered organism in their model training and situations where method architecture was not available to retrain the organism with the examined organism. The methods available for comparison in the imbalanced dataset experiment results are those with imbalanced models or model architecture provided to generate an imbalanced datasets model.

**Table 5:** The results comparison on balanced datasets across the selected organism.

This table shows the results of the Balanced SS prediction accuracy in comparison to the state of the arts for acceptor and donor genome datasets in percent (%). CNNSplice m1 indicates Model 1, CNNSplice m3 indicates Model 3, CNNSplice m4 indicates Model 4. Results highlighted in **bold** black color represent the best result and result highlighted in **bold** red represents the second-best result.

**Table 6:** The results comparison on imbalanced datasets across the selected organism.

This table shows the CNNSplice splice site prediction performance results and its comparison to other methods. We show the prediction accuracy measures, in percent (%), amongst other evaluation metrics performance results. Results highlighted in **bold** black color represent the best result and result highlighted in **bold** red represents the second-best result.

**Table 7:** The performance evaluation metrics for the balanced dataset using model 1.

SpliceSites	Model name	Precision	F1	Recall	Accuracy
Acceptor	Homo sapiens	94.50	94.44	94.44	94.44
	Arabidopsis thaliana	96.31	96.24	96.26	96.26
	Oryza sativa japonica	96.80	96.85	96.80	96.85
	Drosophila melanogaster	94.86	94.74	94.77	94.77
	Caenorhabditis elegans	97.29	97.00	96.69	97.00
					
SpliceSites	Model name	Precision	F1	Recall	Accuracy
Donor	Homo sapiens	96.70	96.63	96.63	96.63
	Arabidopsis thaliana	94.11	94.10	94.10	94.10
	Oryza sativa japonica	94.27	94.23	94.24	94.24
	Drosophila melanogaster	96.16	96.15	96.15	96.15
	Caenorhabditis elegans	97.10	97.04	97.07	97.07

This table shows the CNNSplice splice site prediction performance evaluation results. We show the accuracy, precision, recall and f1 score measures in percent (%) for the balanced acceptor and donor genomic organism datasets.

In conclusion, CNNSplice presents robust DL models for improved SS prediction with comparison to the state-of-the-art methods, namely Splice2Deep [21], GeneSplicer [16], SpliceFinder [24], and DeepSplicer [25] for both balanced and imbalanced acceptor and donor datasets. The plot representation of the results accuracy comparison is shown in Fig. 4*a*, Fig. 4*b*, Fig. 4*c*, and Fig. 4*d*. Based on these findings, we present models for RNA splicing detection and analysis in known or poorly annotated genomic distribution spectrums, which will aid groundbreaking research and clinical experiments. The possibility of uneven genomic dataset distribution in bioinformatics and clinical genetic testing scenarios emphasizes the importance of our imbalanced models.

### 3.4 P-value statistical significance test

The statistical significance of the accuracy results comparison for the balanced datasets presented in Table 8 was determined by employing the nonparametric Friedman test. This test was utilized to ascertain differences among several related groups, and mean ranks were employed to rank the groups. To perform the test, accuracy values were converted to ranks, where the highest accuracy value was assigned a rank of 1, and the lowest accuracy value received a rank equivalent to the number of models being compared. The mean rank for each model across all datasets and SS types was subsequently calculated.

**Table 8:** The Friedman test results comparison on balanced datasets across the selected organism.

Algorithm	Rank	Mean rank
**CNN Splice m1**	**1**	**1.33**
DeepSplicer	2	2.00
CNNSplice m4	3	3.17
Splice2deep	4	4.50
CNN Splice m3	5	4.67
SpliceFinder	6	5.33
GeneSplicer	7	6.67
SpliceRover	8	7.50

This table shows the statistical significance of the Balanced SS prediction accuracy results in comparison to the state of the arts for acceptor and donor genome datasets. The tables show the Algorithm names, Friedman rank position and Mean rank values CNNSplice m1 indicates Model 1, CNNSplice m3 indicates Model 3, CNNSplice m4 indicates Model 4. Results highlighted in **bold** black color represent the best result.

Using the Friedman statistic formula:

(6) $$Q^{2}=\left[\frac{12}{\left(m*k*\left(k+1\right)\right)}\right]*\sum {\left(R^{2}-\left[\frac{\left(k+1\right)}{2}\right]\right)}^{2}\;$$

where:

m = number of models being compared.

k = number of independent tests.

R² = mean rank of the i-th model across all the independent tests.

Based on Friedman test results shown in Table 8, the statistical analysis using the Friedman test yielded a *p-value* less than 0.05, which signifies a significant difference among the models. The ranking based on mean ranks indicates that on average, CNNSplice model 1 performed the best, followed closely by DeepSplicer [25] and CNNSplice model 4. Conversely, Splice2deep [21], CNNSplice model 3, SpliceFinder [24], GeneSplicer [16], and SpliceRover [20] had lower mean ranks, indicating less consistent performance across the various datasets and SS types.

### 3.5 Generalizability test

**Fig. 5:**
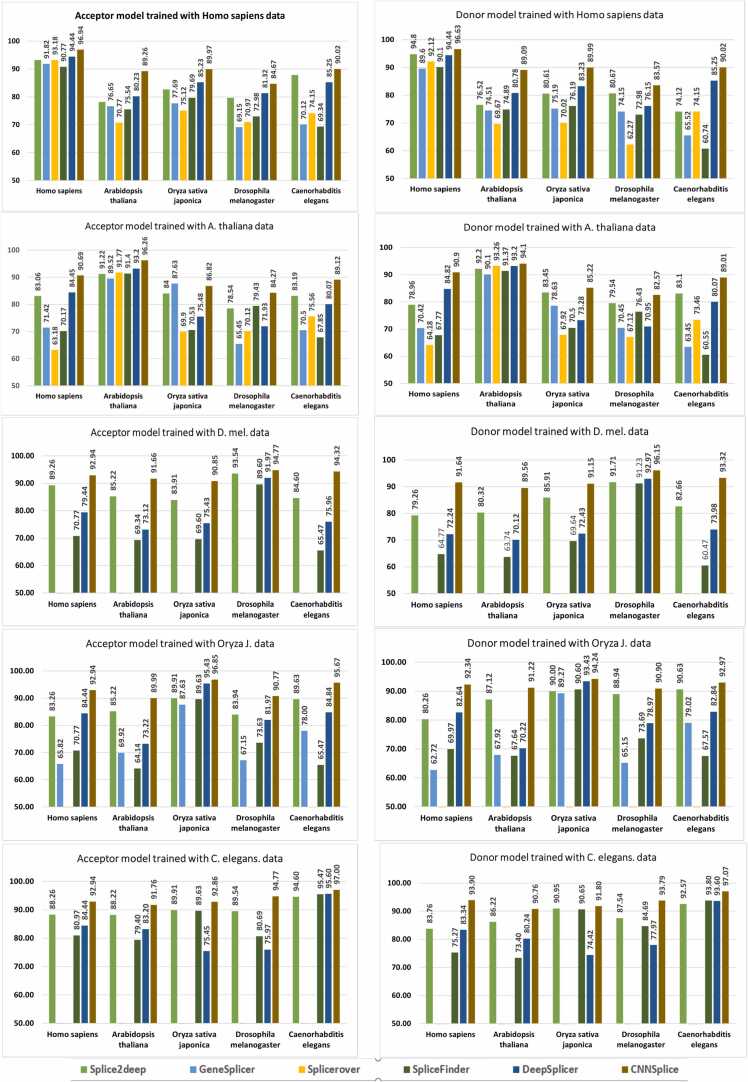
The models’ Generalizability comparison on Balanced datasets across the selected organism. This figure shows the generalization comparison plot for CNNSplice model and other methods. This experiment is done on the selected genome organism dataset with CNNSplice model 1 and the other methods applicable models. No data or bar represent methods with no available models or model architecture for ss testing. We used the balanced dataset to effectively represent all methods involved in the generalization experiments. The Y-axis of the plots represents the prediction accuracy in percentage (%) while the X-axis represents the name of the genome organism used for the experiment.

To assess the generalizability of our approach, we employed an empirical sliding window program that allowed for the efficient selection of local flanking regions and the identification of the SS-containing region based on the dataset under investigation. Furthermore, we enhanced the nonlinearity expression capabilities of our model by enriching its fully connected layer and augmented the proportion of noncanonical SS in the training dataset to improve the model’s ability to detect such SS. This augmentation contributed to an improvement in the model’s generalizability. Each of the organism-generated models underwent the same procedure. For example, a *Caenorhabditis elegans* model was tested utilizing datasets from *Homo sapiens*, *Oryza sativa japonica*, *Arabidopsis thaliana*, and *Drosophila melanogaster*. The results are reported in Fig. 5 and demonstrate CNNSplice’s higher performance over other methodologies, as well as the fact that CNNSplice’s cross-organism test outperformed some other tools trained and tested on the same datasets. For example, CNNSplice balanced acceptor model, trained on *Caenorhabditis elegans* and tested on *Drosophila melanogaster*, has a prediction accuracy of 94.77 %, compared to 86.60 % and 91.97 % for SpliceFinder [24] and DeepSplicer [25] respectively, when trained and tested on *Drosophila melanogaster* balanced acceptor genome organism datasets. This result further validates CNNSplice’s capacity to predict and annotate newly introduced or previously unseen sequenced genome datasets.

### 3.6 Understanding CNNSplice model outputs

**Fig. 6:**
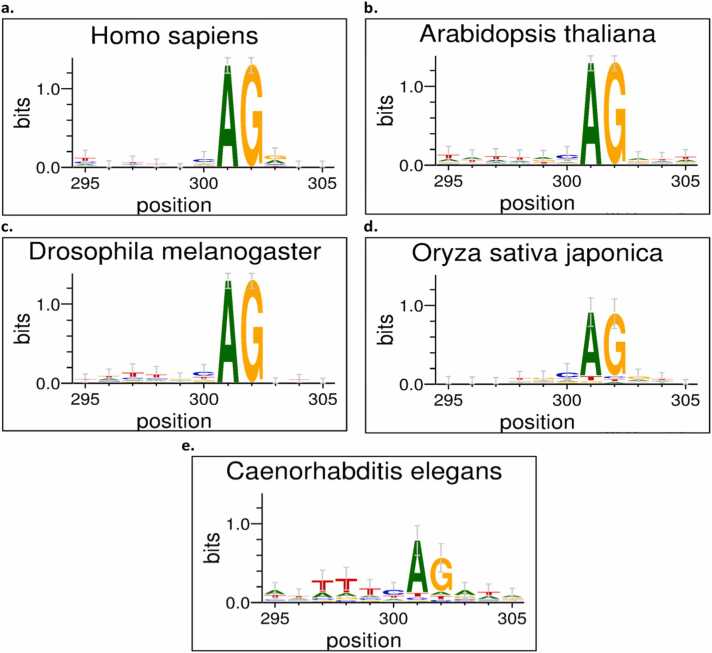
Result validation for acceptor splice site organism. This Figure shows the sequence logo of *the Homo sapiens, Oryza sativa japonica, Arabidopsis thaliana, Drosophila melanogaster, and Caenorhabditis elegans* organisms acceptor splice sites within genomic sequence position 295 and 305. This nucleotide sequence pattern likelihood is depicted by the magnitude of the genomic sequence characters in the motif representation.

**Fig. 7:**
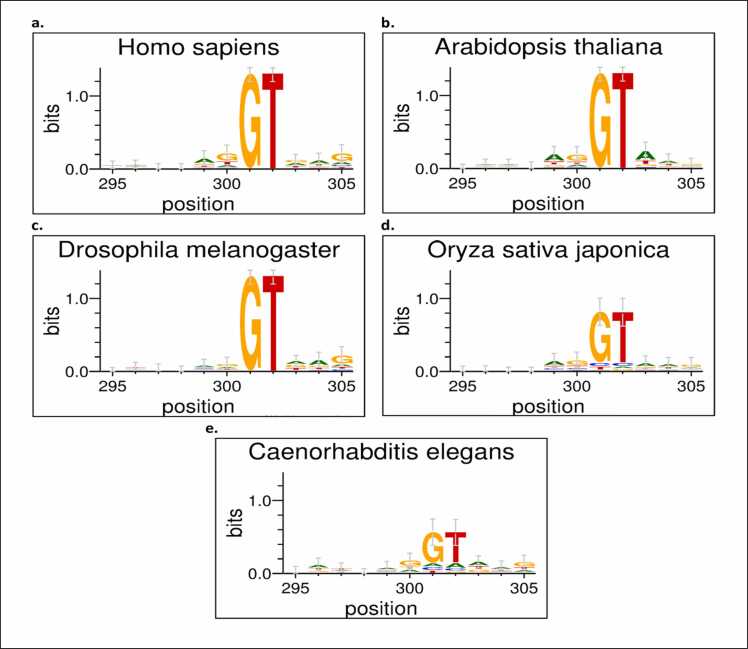
Result validation for donor splice site organism. This Figure shows the sequence logo of the *Homo sapiens, Oryza sativa japonica, Arabidopsis thaliana, Drosophila melanogaster, and Caenorhabditis elegans* organisms donor splice sites within genomic sequence position 295 and 305. This nucleotide sequence pattern likelihood is depicted by the magnitude of the genomic sequence characters in the motif representation.

We determined the average regional contribution and importance score to assess the impact of our feature extraction on the interpretation of our model’s prediction [31]. To accomplish this, we analyzed and determined the feature that powers our model prediction by using motif extraction and visualization. A motif is a collection of subsequences related to a decision process [19]. With the help of Shap [32], we were able to determine the contribution score of each feature instance inside the sequencing window and, as a result, decipher the underlying pattern of our dataset’s characteristics. Shap (SHapley Additive exPlanation) is an approximation approach to explain a machine learning model’s feature output. This tool uses the game theory approach to explain the importance of features in a model. The sequence logo is made up of the contribution score generated from the sequence patterns. WebLogo version 3.7.4 [33] (available at http://weblogo.threeplusone.com/create.cgi) is used to produce the sequence logos. For both the acceptor and donor organism datasets, we randomly picked 100 sequences of each organism. Sequence positions 295–305 are represented by the magnitude of the genomic sequence characters in the motif. As shown *in* Fig. 6 and Fig. 7, AG significantly contributes to the prediction of the acceptor site for each organism dataset, as GT contributes to the prediction of the donor site.

### 3.7 CNNSplice webserver

We have developed a webserver accessible at **http://www.cnnsplice.online** for the purpose of this project. The webserver allows users to input a FASTA sequence or upload a FASTA file, following specific criteria outlined in the "About" Tab. Users must select a pre-trained model for different organisms and provide an email address for notification about a job submission and about the completion of the submission prediction. The completed job will be sent to the user via email as a text file attachment, indicating the presence or absence of a splice site for each sequence in the provided FASTA data using binary data (1 for detection, 0 for no detection). To assist users, we have included a tutorial and user guide with examples accessible through the "Tutorial/Example" Tab on the webserver.

## 4 Conclusion

In this paper, we presented CNNSplice, a powerful biological tool for predicting SS. This bioinformatics solution stems from the biological procedures of RNA splicing that are required for protein synthesis and gene expression. Our method extracts raw genomic features, maps them using one-hot encoding, and feeds them into the CNN architecture as inputs. We selected the best five models based on our five-fold cross-validation, with our model offering superior prediction performance than previous SS prediction tools for both balanced and imbalanced datasets, as stated in the results sections. Furthermore, we showed that the CNNSplice model trained on the organisms utilized in the research may be applied to another species without losing any information, demonstrating its generalizability in annotating new organisms. Based on our findings, we can conclude that CNNSplice offers the following advantages:

(1) CNNSplice is a convenient two-class model that predicts true and false SS in both salient canonical and noncanonical SS.
(2) Our analysis shows that CNNSplice models outperform previous approaches, with our models occupying the Top-1 and Top-2 performance positions across all organisms for both balanced and imbalanced acceptor and donor datasets (Fig. 4). In general, depending on the detection and the organism under consideration, each of these model architectures has its own advantages.
(3) As described in the generalization section, CNNSplice Model 1 provides a robust tool to predict and annotate poorly studied or newly sequenced genomic datasets (Fig. 5). In other words, despite being trained on one genomic organism dataset and evaluated on a different organism dataset, this model yields a high SS prediction accuracy.

Our results show that CNNSplice can extract high-level features and locate SS genomic sequence patterns, which may then be used to infer gene expression information as seen in Fig. 5 for phenotype anomaly and diseases analysis.

## Funding

This work was supported by the start-up funding from the 10.13039/100010174University of Colorado, Colorado Springs to O.O.

## CRediT authorship contribution statement

**Victor Akpokiro:** Methodology, Software, Investigation, Data curation, Analysis, Writing – original draft, Writing – review & editing, Validation. **H. M. A. Mohit Chowdhury:** Software, Investigation, Analysis, Writing – review & editing, Validation. **Samuel Olowofila:** Software, Writing – review & editing, Validation. **Raisa Nusrat:** Software, Writing – review & editing, Validation. **Oluwatosin Oluwadare:** Conceptualization, Methodology, Analysis, Writing – review & editing, Supervision, Resources, Project administration, Funding acquisition.

## Declaration of Competing interest

The authors declare that they have no known competing financial interests or personal relationships that could have appeared to influence the work reported in this paper.

## Data Availability

CNNSplice’s data, models generated, and source code are available as an open-source software at https://github.com/OluwadareLab/CNNSplice. We have included a docker-containerized environment with all dependencies for local install and program runs in the CNNSplice repository.
